# Impact of a virtual Culinary medicine curriculum on fatty acid profiles in medical students

**DOI:** 10.1016/j.metop.2025.100431

**Published:** 2025-12-09

**Authors:** Selina Böttcher, Thomas Ellrott, Miriam Rabehl, Can G. Leineweber, Chaoxuan Wang, Uwe Neumann, Anne Pietzner, Christoph Schmöcker, Karsten H. Weylandt

**Affiliations:** a Medical Department, Division of Gastroenterology, Oncology, Hematology, Rheumatology and Diabetes, ukrb, Brandenburg Medical School, Theodor Fontane, 16816, Neuruppin, Germany; b Faculty of Health Sciences, Joint Faculty of the Brandenburg, University of Technology, Brandenburg Medical School and University of Potsdam, 14469, Potsdam, Germany; c Experimental Lipidology, Brandenburg Medical School, 16816, Neuruppin, Germany; d Institute for Nutrition and Psychology, Georg-August-University Göttingen Medical Centre, Humboldtallee 32, 37073, Göttingen, Germany; e Culinary Medicine Germany e.V., 48341, Altenberge, Germany

**Keywords:** Culinary medicine, Teaching kitchen, Nutrition education for health professionals, Fatty acids, Interprofessional education, Hands-on cooking

## Abstract

**Background:**

Culinary Medicine (CM) has gained increasing popularity as an educational approach to improve nutrition-related competencies among healthcare professionals. Previous studies have demonstrated increased counseling competencies, but also improvements in dietary behaviors among participants, however, objective biomarker-based evidence of such behavioral changes remains scarce. This pilot study aimed to explore preliminary effects of a CM course on biochemical and anthropometric parameters, and to evaluate the feasibility of biomarker assessment among medical students.

**Methods:**

In this exploratory pre-post study, medical students completed a 20-h virtual CM curriculum. Blood samples were collected before and after the course to assess lipid parameters, HbA1c, erythrocyte fatty acid compositions, and body weight.

**Results:**

Of 30 enrolled students, 13 participated in the biomarker assessment. There were slight non-significant decreases in Body Mass Index (−0.08 kg/m^2^, *p* = 0.07) and standard laboratory lipid parameters, including LDL Cholesterol (−0.08 mmol/L, *p* = 0.598) and total cholesterol (−0.12 mmol/L, *p* = 0.493). Significant alterations in erythrocyte fatty acids were detected with a slight increase in saturated fatty acids (+0.78 %, *p* = 0.004) and, in particular, monounsaturated fatty acids (+1.04 %, *p* = 0.004), accompanied by a significant decrease in n-6 polyunsaturated fatty acids (−2.28 %, *p* = 0.003).

**Discussion:**

This pilot study demonstrates the feasibility of conducting biomarker-based evaluations within a CM curriculum and provides preliminary biochemical evidence supporting previous self-reported findings of dietary behavior change. The study illustrates a promising approach for integrating objective outcome measures into CM education and informs the design of future, adequately powered trials.

## Highlights

• First pilot biomarker-based evidence from a Culinary Medicine in medical students.
• Omega-6 fatty acids decreased, and omega-6/omega-3 ratio improved.
• Findings support translating Culinary Medicine into preventive and clinical care.

## 1 Introduction

Poor dietary habits represent a major health hazard, significantly contributing to the onset of various chronic illnesses [1]. In 2019, an estimated 5 million deaths globally were attributable to overweight and obesity [2].

Physicians play a key role in initiating lifestyle interventions, such as nutritional therapy [3]. However, they often lack the required knowledge to support their patients with tailored nutrition counseling [4,5]. An explanation may be an inadequate integration of nutrition education in medical training. In a review by Crowley et al. (2019), the insufficient teaching of nutrition in medical education, across countries, training stages, and teaching formats, was comprehensively highlighted [6].

Culinary Medicine (CM) is a structured educational program that combines theoretical knowledge with practical cooking sessions in a teaching kitchen [7]. It has been developed to address the lack of nutrition-related knowledge among healthcare professionals and is primarily offered at medical schools [8].

Newman et al. recently described CM as: “Culinary medicine programs give medical students the experience necessary to translate nutrition knowledge learned in typical medical school curricula into practical advice. Culinary experiences allow medical students to provide their patients with individualized food and nutrition ideas that are easier to translate into actionable changes than the typical generalities of healthy eating imparted during many patient visits”(8).

This format has demonstrated measurable educational success, including improved nutrition counseling skills [[9], [10], [11], [12], [13]], increased knowledge in nutrition and dietetics [[11], [12], [13]] and greater awareness of the relevance of nutritional therapy in clinical practice [10,11].

CM is now increasingly used in patient education as well [[14], [15], [16]]. Its effectiveness has also been demonstrated in this context: McClure et al. (2025) reported that a CM intervention for individuals with obesity led to an increased cooking frequency, improvements in satiety, snacking behavior and food appreciation and a significant reduction in LDL cholesterol levels [15]. In a study by Sharma et al. (2021), the authors observed a decrease in HbA1c levels and an improvement in dietary behavior among patients with type 2 diabetes following participation in a “Prescription of Healthy Living” CM curriculum [16].

Previous studies on CM have also shown changes in the dietary habits of participants: Razavi et al. (2020) found that participants who had completed the CM Curriculum adhered more closely to the principles of a Mediterranean diet than medical students without such training [10]. Conroy et al. (2004) reported improvements in participants' dietary habits following completion of the courses [17]. Böttcher et al. (2023) also observed changes in participants dietary habits [11]. Helbach et al. (2023) reported that participation in a twelve-part online lecture series on nutritional medicine, without a practical cooking component, improved medical students' self-reported food choices [18]. Nascimento et al. (2024) described in a practical report, that participants in a five-week CM course felt more motivated to improve their lifestyles, including becoming more active in their own kitchens [19]. However, the effects of the eating behaviors of CM on medical students’ eating habits have so far been reported mainly through self-assessments [10,11,17], which are prone to bias and may not accurately reflect true behavioral or physiological change. Objective measures, such as biomarkers can provide stronger evidence of the impact of CM interventions. To date, only patient related CM interventions have included measurable biochemical changes. From a theoretical standpoint, CM may influence metabolic parameters of healthcare professionals through mechanisms including increased consumption on certain foods (e.g. rich in fiber, improved fatty acid quality. However, such hypothesized physiological effects remain largely untested in CM participants.

Given this gap, this pilot study explores the feasibility of conducting biomarker-based assessments within a CM curriculum and aims to generate preliminary data on potential biochemical and anthropometric changes among medical students. By focusing on descriptive biochemical outcomes, this pilot study aims to inform the design of future controlled and adequately powered investigations assessing the objective impact of CM education.

## 2 Materials and methods

### 2.1 Design

This pilot study was conducted at Brandenburg Medical School (MHB) in Germany. Thirteen voluntary participants of two CM courses were included. The study followed a non-randomized, non-controlled observational design. Due to the COVID-19 pandemic, the CM curriculum was implemented in a virtual format.

Data presented in this study derive from the same cohort described in Böttcher et al., 2023 [11], which focused on self-reported outcomes based on questionnaire data. This pilot study uses biochemical markers to evaluate the impact of the intervention in a subset of the cohort.

### 2.2 Participants

Participants were medical students from all semesters who took part in the elective CM courses and chose to participate in the voluntary blood sampling. Recruitment was initiated via an email sent in advance, which included details regarding the opportunity to participate. Written informed consent was obtained from all participants. Inclusion in the study was limited to those who provided signed consent as part of a project investigating blood concentrations of n-6 and n-3 fatty acids in the context of metabolic disease.

### 2.3 Culinary medicine elective

The CM curriculum was offered as an elective and consisted of 28 teaching units, each lasting 45 min. Structured into seven modules, the course was conducted weekly over seven weeks, with each session comprising four lecture hours. Each class included an interactive lecture, discussion of the corresponding recipes, supervised hands-on cooking and tasting. Five to seven recipes were prepared per module, each reflecting the main dietary principles covered in that session. Recipe cards, including step-by-step photo instructions were sent to participants in advance.

The theoretical foundation of the course was formed by the German consensus paper *“manual of nutritional therapy in patient care”* [20]. The course included the following modules: healthy diet, malnutrition, dietary therapy of cardiovascular disease and metabolic disease part I and II, dietary management of gastrointestinal diseases, dietary therapy of kidney disease and dietary therapy for inflammatory rheumatic, orthopedic, neurological and pulmonal diseases.

In the 'Healthy Diet' module, foundational principles from the German Nutrition Society's guidelines were presented, with particular emphasis on fat and oil recommendations, such as favoring olive oil and rapeseed oil [21,22].

An interdisciplinary team led the course, including a registered dietitian and medical student tutors trained in the Culinary Medicine program. Due to the Covid-19 pandemic, the course content was provided online using Cisco Webex (Version 45.7.1.32733) in an interactive format with video and audio participation. To maintain interactivity, each course was limited to 15 medical students. The curriculum has been described in detail by Böttcher et al. (2023) [11].

### 2.4 Evaluation tools and blood samples

To evaluate the effectiveness of the intervention, a voluntary pre- and post-course standardized self-assessment questionnaire was used, including the following categories: counseling competencies, personal attitudes towards nutrition counseling in clinical practice, nutrition knowledge, WHO-5, and eating habits. The results have been published previously by Böttcher et al., 2023 [11]. Additionally, the first two CM courses at MHB offered participants the option to take part in a program that would assess the potential changes of essential fatty acids in two blood panels. An initial blood sample was obtained one week before the course commenced, followed by a second sample collected within two weeks after the final session, roughly two months after the first assessment. For standard laboratory analyses, a 2.7 mL ethylenediaminetetraacetic acid (EDTA) tube (Sarstedt, Germany) and a 7.5 mL serum collection tube (Sarstedt, Germany) were collected from each participant. For the fatty acids analysis in red blood cells, one 7.5 mL EDTA tube (Sarstedt, Germany) was required. EDTA blood samples were separated into plasma and erythrocytes by centrifugation (3500 rpm, 4 min, 4 °C), kept in cryogenic vials and stored at −80 °C until gas chromatography (GC) analysis. All participants were instructed to attend the blood collection session in a fasting state. For one participant, only a single small EDTA sample could be obtained, which was sufficient for erythrocyte fatty acid analysis but insufficient for standard laboratory measurements. Consequently, analyses of standard parameters such as triglycerides, LDL and HDL-cholesterol were performed with data from 12 participants (n = 12), whereas erythrocyte fatty acids were analyzed in all 13 participants (n = 13).

### 2.5 Analysis of the blood samples

Standard laboratory parameters were analyzed using the routine laboratory at University Hospital Ruppin-Brandenburg. Fatty acids from erythrocyte samples were extracted and derivatized using an adapted borontrifluoride (BF_3_) methylation protocol, as previously described [[23], [24], [25]]. In short, fatty acids from 50 μL erythrocytes per sample were extracted and analyzed by GC. GC was performed on a 7890B GC System (Agilent Technologies) with a HP88 Column and nitrogen as the carrier gas. Fatty acid concentrations from blood cells are presented as percent [%] of total fatty acid content. The following fatty acids were detected and analyzed: myristic acid (14:0), palmitic acid (16:0), palmitoleic acid (16:1n-7), stearic acids (18:0), oleic acid (18:1n-9), linoleic acid (18:2n-6), eicosanoic acid (20:0), docosanoic acid (22:0), dihomo-gamma linolenic acid (20:3n-6), arachidonic acid (20:4n-56, eicosapentaenoic acid (20:5n-3), lignoceric acid (24:0), nervonic acid (24:1n-9), docosapentaenoic acid (22:4n-6), docosapentaenoic acid (22:5n-3), docosahexaenoic acid (22:6n-3).

### 2.6 Data collection and analysis

Data collection and statistical analyses were conducted using Microsoft Excel (16.75.2), and IBM SPSS Statistics (28.0.1.0). Statistical calculations, t-tests, Wilcoxon tests and graphical visualizations were performed using IBM SPSS Statistics (28.0.1.0) and GraphPad Prism 10 10.2 (171).

Data were tested for normal distribution using the Shapiro-Wilk test and the difference between the pre- and post-intervention was compared accordingly using a paired two tailed *t*-test or Wilcoxon matched pairs signed rank test. Statistical significance was defined at p < 0.05. No correction for multiple comparisons was applied, as this was an exploratory pilot study aimed at testing the effect on potentially harmful – as potentially pro-inflammatory – n-6 PUFAblood content, and on hypothesis generation.

Effect sizes were calculated using Cohen's *dz*. The following thresholds were applied: No effect at *d* < 0.2, a small effect for *d* = 0.2–0.49, a medium effect for *d* = 0.5–0.79, and a large effect at *d* ≥ 0.8 [26]. Data of the two cohorts were collected between April 2021 and June 2021, as well as July 2021 and September 2021. Although the manuscript was submitted in 2025, all data were collected in 2021.

### 2.7 Ethical approvement

The blood examination study was approved by the institutional ethics committee of the Brandenburg Medical School, Theodor Fontane, Nr. Z02-20170508.

## 3 Results

### 3.1 Participants

A total of 30 students participated in the two CM courses. Of these, 13 participants (43 %) were included in this observational study. In one case, only one small EDTA sample could be obtained for the standard laboratory analysis. Therefore, the analyses of standard parameters such as triglycerides, LDL- and HDL-cholesterol were performed with data from 12 participants, while the erythrocyte fatty acids were analyzed in 13 participants. Detailed participant characteristics can be found in Table 1.

**Table 1:** Baseline characteristics of participants.

Mean Age	25.5 (±5.5)
Gender	
Female (n)	12
Male (n)	12/1
Mean Semester	4.4 (±5.3)
BMI	22.29 kg/m2
BMI 18.5–24.9 kg/m2	100 %
BMI >25 kg/m2	0 %
Race	
non- Hispanic white	100 %
Other race	0 %

Foot notes: BMI = Body Mass Index.

### 3.2 Body weight and laboratory parameters

**Fig. 1:**
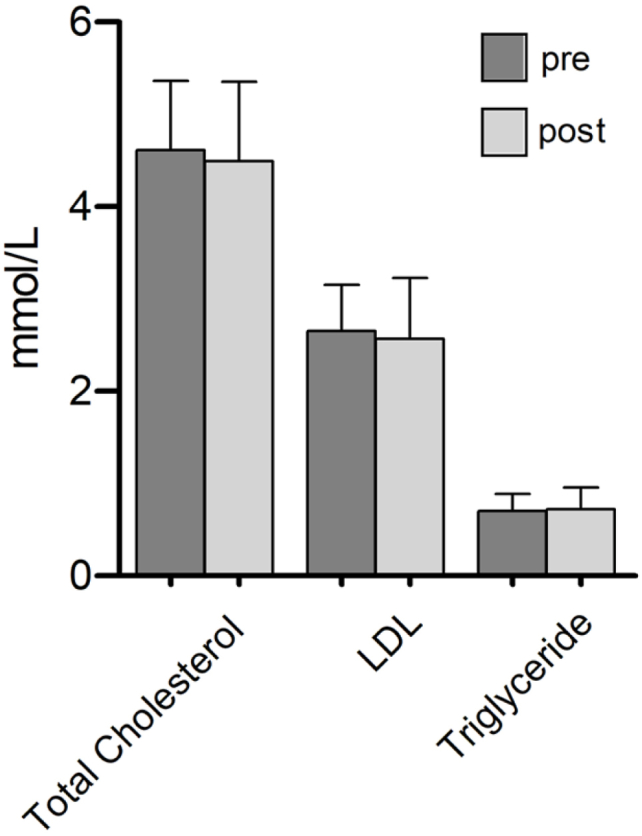
Pretest and Posttest changes in routine laboratory parameters in medical students in serum after a Culinary Medicine course intervention in Neuruppin, Germany, 2021. Foot notes: Mean +SD, n = 12. After testing for normality using the Shapiro-Wilk test, statistical analyses were performed using paired two-tailed Student's t-test. Changes were considered significantly different when indicated: ∗<0.05. LDL = low density lipoprotein cholesterol.

Slight reductions in LDL and total cholesterol were observed between the pre- and post-course measurements (see Fig. 1). However, these changes did not reach statistical significance. LDL cholesterol decreased from 2.65 mmol/L to 2.56 mmol/L (*p* = 0.598). Total cholesterol decreased from 4.61 mmol/l to 4.49 mmol/L (*p* = 0.493). Triglyceride levels stayed essentially the same, rising from 0.70 mmol/L to 0.72 mmol/L (*p* = 0.712). In addition, there was only a slight non-significant change in increase in HbA1c, rising from 4.93 % to 4.98 % (*p* = 0.429).

A reduction in body weight from 67.07 kg to 66.57 kg was observed, which showed a trend toward significance (*p* = 0.059), though not statistically significant. Body mass index (BMI) thus also decreased slightly from 22.29 kg/m^2^ to 22.21 kg/m^2^ (seeFig. 2).

**Fig. 2:**
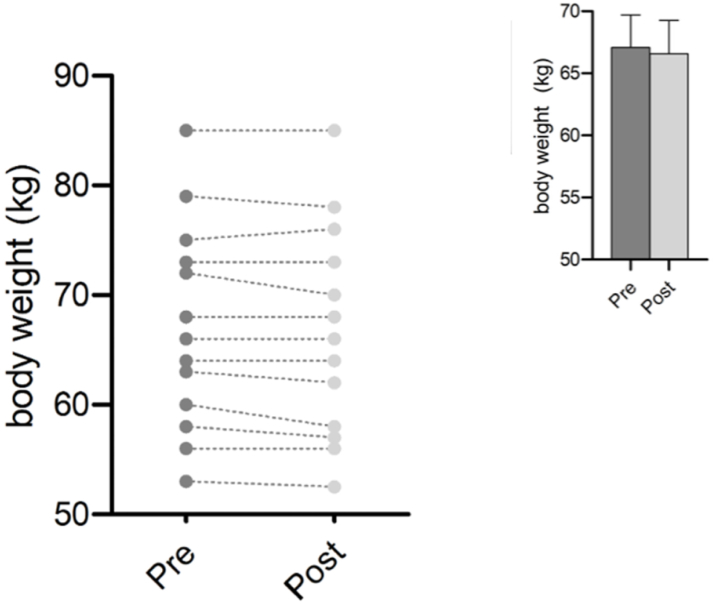
Pretest and Posttest changes in body in medical students in serum after a Culinary Medicine course intervention in Neuruppin, Germany, 2021. Foot notes: Mean +SD, n = 13. After testing for normality using the Shapiro- Wilk test, statistical analyses were performed using paired two-tailed Student's t-test. Changes were considered significantly different when indicated: ∗<0.005. Kg = kilogram.

### 3.3 Analysis of fatty acids

**Fig. 3:**
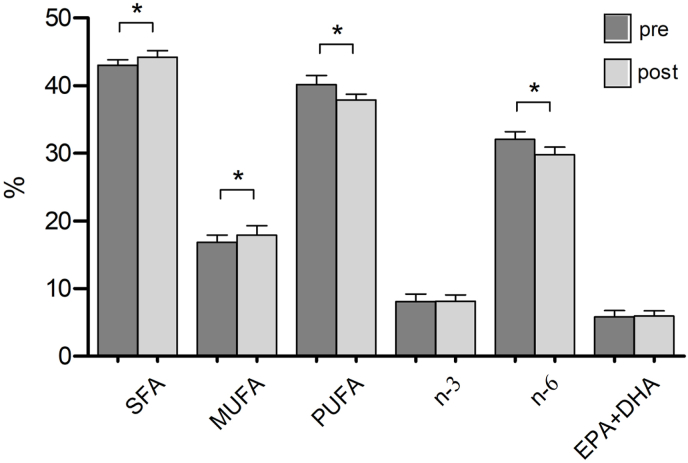
Pretest and Posttest changes in erythrocyte fatty acid concentrations [%] after a Culinary Medicine course intervention in Neuruppin, Germany, 2021. Foot notes: Mean ± SD, n = 13. Statistical analyses were performed using paired two-tailed Student's t-test and Wilcoxon matched pairs signed rank test. Changes were considered significantly different when indicated: ∗<0.05. SFA= Saturated fatty acids; MUFA = monounsaturated fatty acids; n-3 = Omega-3 fatty acid; n-6 = Omega 6 fatty acids; EPA + DHA = eicosapentaenoic acid + docosahexaenoic acid.

**Fig. 4:**
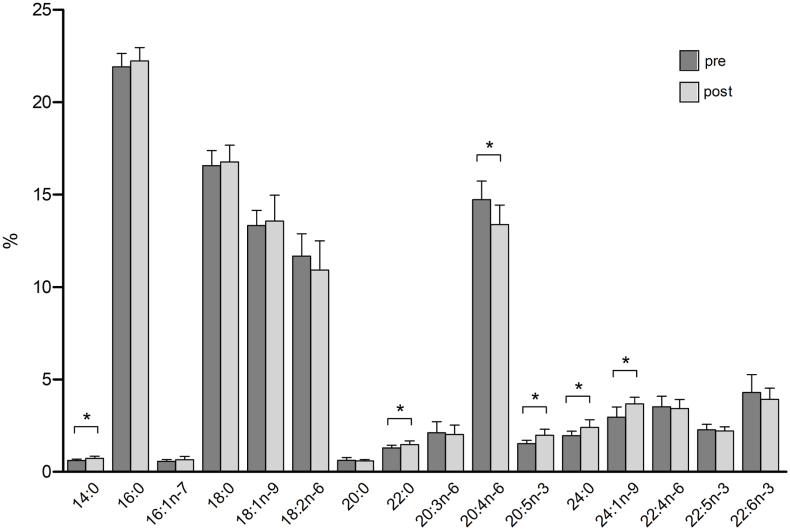
Pretest and Posttest changes in erythrocyte single fatty acid concentrations [%] after a Culinary Medicine course intervention in Neuruppin, Germany, 2021. Foot notes: mean ± SD, n = 13. Statistical analyses were performed using paired two-tailed Student's t-test. Changes were considered significantly different when indicated: ∗<0.05.

In contrast, even with this limited intervention, highly significant changes were observed in several erythrocytes fatty acid concentrations between the pre- and post-course measurements. Saturated fatty acids (SFA) increased significantly from 42.99 % to 44.21 % (*p* = 0.004) and monounsaturated fatty acids (MUFA) increased from 16.86 % to 17.90 % (*p* = 0.004). In contrast, polyunsaturated fatty acids (PUFA) decreased significantly from 40.14 % to 37.88 % (*p* = 0.003). And while n-3 polyunsaturated fatty acids (n-3 PUFA, comprised of eicosapentaenoic acid (EPA), docosapentaenoic acid (DPA) and docosahexaenoic acid (DHA), remained nearly unchanged, n-6 polyunsaturated fatty acids (n-6 PUFA) decreased significantly from 32.03 % to 29.75 % (*p* < 0.001) (see Table 2). Consequently, the n-6/n-3 ratio decreased from 4.03 to 3.72, but did not reach statistical significance (*p* = 0.101). The combined content of long-chain n-3 PUFA EPA and DHA showed a slight increase from 5.82 % to 5.91 %, but this change was not statistically significant (*p* = 0.669) (see Fig. 3, Fig. 4).

**Table 2:** Pretest and Posttest changes in erythrocyte fatty acid concentrations [%] after a Culinary Medicine course intervention in Neuruppin, Germany, 2021.

	Mean (pre) ± SD	Mean (post) ± SD	Mean difference	*t-* value	Cohen's *dz*	*p*-value
SFA (%)	42.99 (0.827)	44.21 (0.977)	1.213	−0.360	1.213	∗0.004
MUFA (%)	16.86 (1.059)	17.90 (1.377)	1.046	−3.586	1.052	∗0.004
n-3 (%)	8.10 (1.098)	8.12 (0.923)	0.026	−0.104	0.927	0.919
n-6 (%)	32.03 (1.161)	29.75 (1.167)	−2.286	5.891	0.991	∗<0.001
EPA + DHA (%)	5.82 (0.928)	5.91 (0.780)	0.086	−0.438	0.712	0.669

Foot notes: Mean ± SD, n = 13. After testing for normality using the Shapiro- Wilk test, statistical analyses were performed using paired two-tailed Student's t-test. Changes were considered significantly different when indicated: ∗<0.005. Cohen's dz was calculated as the mean difference divided by the standard deviation of the paired differences.

SFA= Saturated fatty acids; MUFA = monounsaturated fatty acids; n-3 = Omega 3 fatty acid; n-6 = Omega 6 fatty acids; EPA + DHA = eicosapentaenoic acid + docosahexaenoic acid.

## 4 Discussion

Changes in dietary habits among participants of CM courses have been described in several studies [10,11,17]. However, these changes were based primarily on self-assessment using questionnaires.

This pilot study explored the feasibility of integrating objective biochemical assessments into a CM curriculum and provided preliminary evidence for short-term dietary behavior changes among medical students.

Although medical students may be considered relatively health-conscious, they receive very limited formal nutrition education. Therefore, substantial knowledge and counseling gaps persist despite this presumed awareness. CM specifically aims to address these gaps through an applied, hands-on teaching approach, which supports the suitability of this population for an initial mechanistic pilot study.

In this study, participants did not receive explicit dietary recommendations. However, the first module introduced the fundamentals of a healthy diet based on the guidelines of the German Nutrition Society for healthy adults [22]. These also included an appropriate selection of dietary oils and fats. Olive oil and canola oil were emphasized as the preferred fat source, with linseed oil as a complementary option [21,27]. Because these dietary patterns are also beneficial in the treatment of various nutrition-related diseases, they were repeatedly highlighted and discussed during the course. Participants were encouraged to ask nutrition-related questions throughout the sessions, which they frequently used to address personal dietary concerns.

The changes observed in the fatty acid profiles could be consistent with the hypothesis that CM participation may be associated with short-term dietary adjustments. A particularly noteworthy finding is the significant reduction in n-6 fatty acids, which aligns with the dietary principles taught. Vegetable oils such as sunflower seed oil, wheat germ oil and safflower oil are high in polyunsaturated fatty acids and typically exhibit an unfavorable n-6 to n-3 ratio [28]. According to the recommendations of the German Nutrition Society, rapeseed oil and olive oil are promoted as preferred fats during the course and were primarily featured in the recipes [21,27]. In baking recipes oil was used instead of butter. Both oils are rich in MUFA, in particular oleic acid. Canola oil presents favorable ratio of fat composition concerning SFA, MUFA and PUFA, as well as a beneficial n-6/n-3 ratio [21,28].

The assumption of increased rapeseed and olive oil use is one possible explanation for the reduction in PUFA and the improvement of the n-6/n-3 ratio [28]. These findings suggest that participants likely incorporated course content immediately into their personal cooking and food preparation routines.

Since n-6 fatty acids are also abundant in animal fats and highly processed foods, further dietary changes, beyond the oil selection are possible. This is supported by the observed slight reduction in body weight, indicating potential changes in dietary behavior. This assumption is corroborated by the results from a previously published analysis of questionnaire data collected from the same cohort: Students reported a significant reduction in the consumption of nutritionally less favorable foods such as sweets, butter, red processed meat, caloric beverages and alcohol (*p* ≤ 0.001), as well as a significant decrease in the consumption of sweets (*p* = 0.006) and red processed meat (*p* = 0.012) [11].

The modest changes observed in the lipid parameters (e.g. LDL, triglycerides) must be interpreted in the context of the study population, which consisted of metabolically healthy young medical students. In contrast, the study by McClure et al. (2025) demonstrated a significant reduction in LDL cholesterol levels among patients with obesity, however it should be noted that the observation period in that study was longer (16 weeks) compared to the current study (8 weeks) [15].

However, the interpretation of the results, particularly the increase in SFA and MUFA alongside a decrease in PUFA, requires caution. While an increased use of olive and rapeseed oil would not typically be expected to raise SFA levels, several alternative explanations may account for this finding. First, participants may have reduced their intake particularly of n-6 PUFA-rich seed oils, which could shift the relative fatty acid distribution towards higher SFA and MUFA proportions without an actual increase in SFA intake. Second, changes in the consumption of processed foods or animal products, which often contain hidden fats, may have influenced the overall fatty acid pattern. Third, the short observation window may not fully capture the kinetics of fatty acid incorporation into erythrocyte membranes, especially for PUFA.

Overall, the exploratory nature of this pilot study does not allow conclusions about causality but provides useful signals for future research.

Limitations of the study include the small overall sample size, the gender-imbalance and the incomplete follow-up data collection, all of which reduce the statistical power and limit the generalizability of the findings. To enhance the generalizability of the findings, a larger sample size and the inclusion of a control group would be necessary. As participation in the study was voluntary, the potential for selection bias cannot be excluded, as students with a higher interest in nutrition or healthier lifestyles may have been more likely to enroll. Given the exploratory pilot design, no correction for multiple comparisons was performed. Thus, the biochemical results should be interpreted with caution. Moreover, the timing of the data collection must be considered. Although the manuscript was submitted at a later stage, the study itself was conducted in 2021. The first round of data collection coincided with a nationwide COVID-19 lockdown, while by the time of the second round most of the restrictions had been lifted, potentially affecting eating behavior and physical activity levels [29,30].

A structured evaluation of the frequency with which participants continued using the provided recipe cards outside the course sessions was not performed, which may limit conclusions regarding the long-term implementation of the dietary recommendations taught during the Culinary Medicine modules.

## 5 Conclusion

In summary, this study provides preliminary objective evidence supporting the self-reported findings of previous CM research [11]. Even though, the biochemical changes were modest, they provide a valuable proof of concept that future studies can build upon for participants, but also patients. The results underline the effectiveness and relevance of CM in preparing future healthcare professionals to serve as role models for their patients [31], especially given the well-established connection between physicians’ own lifestyles and their implementation of lifestyle medicine in clinical practice [[32], [33], [34], [35]].

## CRediT authorship contribution statement

**Selina Böttcher:** Writing – original draft, Visualization, Project administration, Formal analysis, Data curation. **Thomas Ellrott:** Writing – review & editing, Supervision, Project administration, Funding acquisition, Conceptualization. **Miriam Rabehl:** Methodology, Data curation. **Can G. Leineweber:** Methodology, Data curation. **Chaoxuan Wang:** Methodology, Data curation. **Uwe Neumann:** Supervision, Funding acquisition, Conceptualization. **Anne Pietzner:** Writing – review & editing, Methodology. **Christoph Schmöcker:** Supervision, Data curation, Conceptualization. **Karsten H. Weylandt:** Writing – review & editing, Supervision, Funding acquisition, Formal analysis, Conceptualization.

## Ethical approval

The blood examination study was approved by the institutional ethics committee of the Brandenburg Medical School, Theodor Fontane, Nr. Z02-20170508.

## Data availability statement

The data underlying this article will be shared on reasonable request to the corresponding author.

## Declaration of generative AI and AI- assisted technologies in the writing process

We used ChatGPT in order to improve langue and help with translation. After using it, we reviewed and edited the content as needed and take full responsibility for the content of the published article.

## Conflict of interest

The authors declared no potential conflicts of interest with respect to the research, authorship, and/or publication of this article.
